# Predictor Factors for Chronicity in Immune Thrombocytopenic Purpura in Children

**DOI:** 10.3390/children10060911

**Published:** 2023-05-23

**Authors:** Vasile Eduard Rosu, Solange Tamara Roșu, Anca Viorica Ivanov, Iuliana Magdalena Starcea, Violeta Streanga, Ingrith Crenguta Miron, Adriana Mocanu, Ancuta Lupu, Vasile Valeriu Lupu, Cristina Gavrilovici

**Affiliations:** 1 Pediatrics, “Grigore T. Popa” University of Medicine and Pharmacy, 700115 Iasi, Romaniaadriana_baltag@yahoo.com (A.M.); valeriulupu@yahoo.com (V.V.L.);; 2 Nursing, “Grigore T. Popa” University of Medicine and Pharmacy, 700115 Iasi, Romania

**Keywords:** immune thrombocytopenia, chronic thrombocytopenic purpura, child, risk factors for chronicity

## Abstract

(1) Background: Immune thrombocytopenia (ITP) is an acute autoimmune blood disorder that is the main cause of thrombocytopenia in children. It is characterized by a decrease in platelets below 100 × 10^9^/L, and limited evolution with severe complications such as intracranial hemorrhage. The chronic form is defined by the persistence of thrombocytopenia more than 12 months after diagnosis. (2) Methods: We performed a retrospective study over a period of 10 years (1 January 2011–31 December 2020) at the Emergency Clinical Hospital for Children “Sf. Maria”, Iasi. The aim of the study was to describe the clinical characteristics and to determine the prognostic factors in immune thrombocytopenia in children. (3) Results: In this study we included 271 children with ITP, comprising 123 females (45.4%) and 148 males (54.6%). The remission rate was higher in males, being 68.9% compared to 56.1% in females. Children with ITP under 9 years of age had a higher remission rate. Children with a platelet count > 10 × 10^9^/L at diagnosis had a higher likelihood-of-remission rate compared to patients who presented initial platelet count below this value. (4) Conclusions: The risk factors highly suggestive for chronicity are: age at diagnosis, female sex, and the number of platelets at the onset of the disease.

## 1. Introduction

Immune thrombocytopenia (ITP) in children is an acute autoimmune blood disorder, characterized by a decrease in platelets below 100 × 10^9^/L without other secondary causes of thrombocytopenia. The incidence of childhood immune thrombocytopenia varies between 1.6 and 6 per 100,000 children [1]. Clinical signs of ITP include: petechiae, ecchymoses, hematomas, epistaxis, and hematuria. In severe cases, intracranial hemorrhage (the most serious complication, but also the rarest, occurring ~0.5% in children and 1.5% in adults), gastrointestinal hemorrhage, and genito-urinary hemorrhage may occur [2]. The frequency of severe bleeding episodes is approximately 1 in 800 cases (0.2–0.9% of all cases) [3]. Most children with ITP achieve spontaneous remission in the first 6 months; 40% of children present the persistent form of the disease (the duration of thrombocytopenia lasts between 3 and 12 months, and includes patients who do not show spontaneous remission or have not obtained a complete response to therapy) and 10–20% develop the chronic form (the presence of thrombocytopenia for at least 12 months) [4]. Chronic ITP is defined as the persistence of thrombocytopenia with platelet values < 100 × 10^9^/L for 12 months [1,5]. The scope of this retrospective study is to describe the clinical characteristics of ITP at onset; to evaluate the evolution, treatment options, and complications; and to determine the risk and prognostic factors in immune thrombocytopenia in children.

## 2. Materials and Methods

We retrospectively reviewed the medical records of the children diagnosed with ITP in the registry of the Emergency Clinical Hospital for Children “Sf. Maria”, Iasi, over a period of 10 years (1 January 2011–31 December 2020). In the study we included all children under 18 years diagnosed with immune thrombocytopenia. All cases of secondary thrombocytopenia (thrombocytopenia from genetic syndromes such as TAR syndrome, neoplasia, drug-induced thrombocytopenia, and other autoimmune diseases) were excluded. We defined the remission of ITP as a platelet count ≥ 100 × 10^9^/L for at least 2 months. This study was conducted in accordance with the Declaration of Helsinki, and approved by the Institutional Review Board of our hospital (No. 679 from 9 January 2020).

## 3. Results

**Figure 1:**
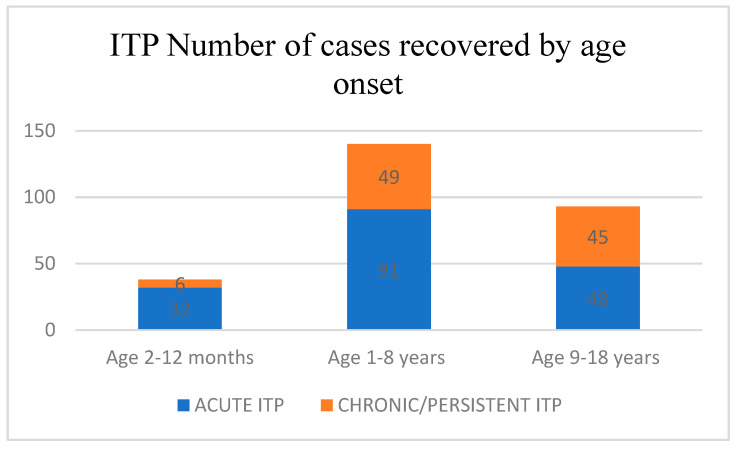
Age of the disease onset for the first category (based on Donato model).

The data were collected in a Microsoft Excel database from the medical records of our hospital. We used the following data: age, sex, the number of platelets at admission, the type of purpura (“wet purpura”/“dry purpura”), and treatment plan applied. The patients were monitored at 1, 3, 6, and 12 months. In this study we used descriptive analysis and the chi square test. In total, 271 children with ITP were included, of which 123 were females (45.4%) and 148 were males (54.6%). The remission rate was higher in males, being 68.9% compared to 56.1% in females. The threshold level of significance was 0.05. The obtained *p*-level of significance was 0.029 (*p* < 0.05). The ratio of the chances of recovery was in favor of the male sex (males have a 1.735-times higher chance of recovery compared to the female sex). Regarding the analyses by age groups at the onset of the disease, we divided the studied group into two categories. For the first category, we used the Donato model [6] and therefore, obtained three age groups: 2–12 months; 1–8 years; and 9–18 years (Figure 1).

For the first two age ranges (1–12 months and 1–8 years) the remission rate was 84.2% and 65.0%, respectively. For the third age range (9–18 years) the remission rate was 51.6%. The threshold value was 0.05. Children with ITP under 9 years of age had a higher remission rate then the children aged over 9 years with *p* = 0.002 (*p* < 0.05), which is highly statistically significant. The prevalence of chronic/persistent ITP between the three groups was low for the 2–12-month group, being only 15.78%; for the 1–8-year-old group it was higher, being 35%, and for the 9–18-year-old group the evolution towards the chronic form was almost 50% (48.38%).

**Figure 2:**
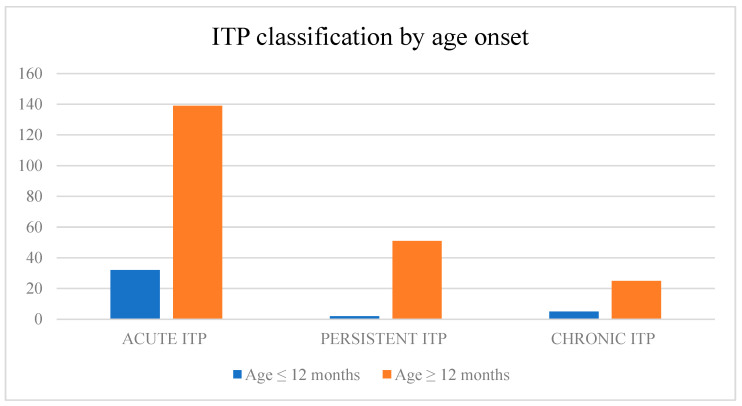
Age at disease onset in the second category (<12 months of age, >12 months of age).

For the second category, we divided the studied group into two age groups: under 12 months of age at disease onset and over 12 months of age after disease onset (Figure 2). The remission rate for the age group under 12 months of age at disease onset was 84.2%, compared with 59.7% in the age group over 12 months. Children with ITP under 12 months of age at disease onset had a higher remission rate with *p* = 0.004 (*p* < 0.05). Children aged <12 months at diagnosis had a higher likelihood (OR: 3.607)-of-remission rate compared to patients over 12 months of age at disease onset.

We performed a statistical analysis in the age groups of 2–12 months and 9–18 years regarding the baseline platelet count being <10 × 10^9^/L at disease onset. Patients aged 2 to 12 months with a baseline platelet count < 10 × 10^9^/L showed a higher remission rate of 83.3% compared to a remission rate of 56.1% for patients aged 9–18 years with a baseline platelet count < 10 × 10^9^/L (Table 1).

We analyzed the remission rate by baseline platelet count at disease onset. For this reason, we divided the study group into two categories: in the first category, the patients presented with an initial platelet count < 10 × 10^9^/L, and in the second category, the initial platelet count was >10 × 10^9^/L. The remission rate was higher for the first category, being 69.4% versus 58.1% for the second category, and *p* = 0.053, which shows minimal significant results (0.05 < *p* < 0.1).

**Figure 3:**
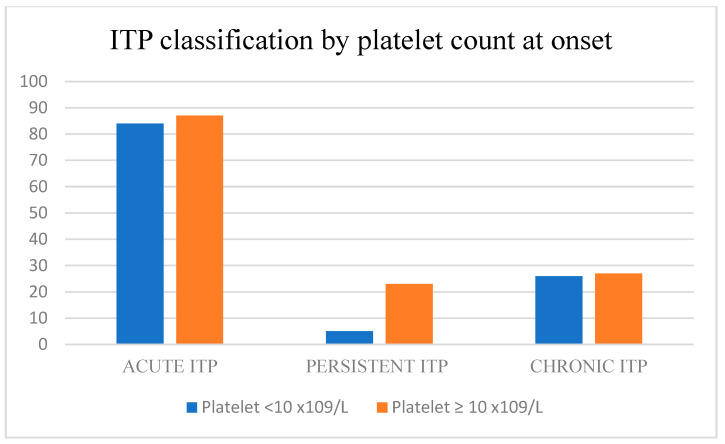
Platelet count at disease onset.

Children with a platelet count > 10 × 10^9^/L at diagnosis had a higher likelihood (OR: 1.644)-of-remission rate compared to patients who presented an initial platelet count < 10 × 10^9^/L at diagnosis (Figure 3).

We analyzed the remission rate according to the type of purpura on admission. Out of 271 patients with ITP, 29.5% had wet purpura and 70.5% had dry purpura. Wet purpura was found in 58 cases of acute ITP, 21 cases of persistent ITP, and 7 cases of chronic ITP. When we analyzed the remission rate according to the presence of wet purpura at diagnosis, no statistical significance was found; *p* = 2.238, (*p* > 0.05).

From the total children who presented with dry purpura at the first admission, 113 were diagnosed with acute ITP, 44 with persistent ITP, and 28 with chronic ITP. No statistical correlation was found between the presence of dry purpura at diagnosis and the form of ITP; *p* = 2.259 (*p* > 0.05).

The treatment regimen consisted of cortisone therapy in 168 cases (62.0%), a combination of corticosteroid therapy and intravenous immunoglobulins (IVIG) in 67 cases (24.7%), and IVIG in 10 cases (3.7%), whereas 4 patients (1.5%) were splenectomized and 22 (8.1%) received no treatment.

In our study, out of 271 patients, 171 (63.1%) were diagnosed with the acute form of ITP, 65 (24.0%) with persistent ITP, and, 35 (12.9%) with chronic ITP. An analysis of persistent and chronic ITP cases revealed the following: the female sex is more frequently affected compared to the male sex (53.1% vs. 46.9%), with there being a greater number of hospitalizations (>6) in female patients compared to male patients (69.4% vs. 55.8%; *p* = 0.032, *p* < 0.05). The risk of having multiple hospitalizations for a female patient with the chronic or persistent form of ITP is 2.8 times higher than for a male patient.

Out of all patients with persistent and chronic ITP, 64.6% of females and 35.4% of males received only cortisone therapy. The double association of cortisone therapy + IVIG was used in 64% of males and 36% of females; *p* = 0.020, *p* < 0.05. In patients with persistent or chronic ITP, the likelihood of receiving only cortisone therapy is 3.24 times greater for females.

We also observed that for patients 12 months or older, corticosteroid treatment was more prevalent. In contrast, for children under 12 months, the proportion of those treated with cortisone therapy was equal to those treated with both corticosteroids and IVIG (Table 2).

Notably, there was a significant rise in the proportion of patients who did not require treatment after reaching 1 year of age. Since our data are categorical, we used the non-parametric Kruskal–Wallis test (Table 3), a rank-based test, to compare the three independent groups (corticosteroids, corticosteroids + IVIG, and no treatment). We excluded the IVIG and splenectomy groups from our analysis.

## 4. Discussion

### 4.1. History of ITP

Purpura is a Latin word meaning “purple”. As a clinical symptom, purpura was recognized by the ancient Greeks and Romans (Hippocrates and Galen) who described it as a red “eminence” associated with plaques (pestilential fevers) [7]. In the 10th century, the Muslim philosopher and physician Abu Ali Al-Hussain Ibn Abdullah Ibn Sina (Avicenna) (980–1037) described a chronic form of purpura that was highly suggestive of the diagnosis of ITP [8]. In 1735, the German physician and poet Paul Gottlieb Werlhof (1699–1767) provided a classic description of ITP in a 16-year-old girl with cutaneous and mucosal bleeding and termed the disease “morbus maculosus haemorrhagicus”, which was later known as Werlhof’s disease [3,9]. In 1916, as a medical student in Prague, Paul Kaznelson hypothesized that the spleen was the site of platelet destruction and convinced his tutor, Professor Doktor Schloffer, to perform the first splenectomy in chronic ITP. The splenectomy was followed by a significant increase in platelet count [8]. The immune nature of ITP was demonstrated for the first time in 1951 by William J Harrington, and in the same year, Evans was able to identify a plasma factor as an antiplatelet antibody [10]. Over time, various therapies have been used for chronic ITP, starting with cortisone therapy (Wintrobe), intravenous immunoglobulins (Imbach), anti-D globulin (Salama), rituximab, and recently, thrombopoietin agonists (TPO) have been introduced [10].

### 4.2. Pathophysiology

The pathophysiology of ITP is still not well understood, and several mechanisms have been proposed, including the formation of autoantibodies, and the involvement of cytotoxic T cells targeting platelets and/or megakaryocytes [2]. Autoantibodies which target platelet surface glycoproteins (GPs) have been shown to be the main factors responsible for platelet clearance [11]. About 70–80% of subjects have autoantibodies against GPIIbIIIa (integrin αIIbβ3), 20–40% of subjects have autoantibodies against the GPIb complex, and some subjects have autoantibodies against both GPs [12]. The destruction of platelets after autoantibody binding was thought to take place in the spleen due to the binding of the Fc portion of immunoglobulins on the surface of platelets to the specific receptors FcγRIIa and FcγRIIIa on tissue macrophages of the splenic reticuloendothelial system. Consequently, the first-line therapies such as IVIG and anti-Rh(D) target these Fc- and FcγR-dependent mechanisms to restore platelet counts [13]. An asialoglycoprotein counter-receptor predominantly expressed on hepatocytes, the Ashwell–Morell receptor (AMR), was recently reported to mediate the clearance of desialylated GP, which was linked to the maintenance of normal platelet turnover through platelet glycosylation status [14]. This leads to Fc-independent platelet clearance in the liver mediated by AMR. Most importantly, this pathway is susceptible to sialidase inhibition. Because IVIG is a limited and very expensive therapeutic resource, and despite the important side effects of steroids, sialidase inhibitors could be an innovative therapy for patients unresponsive to conventional initial treatments and splenectomy [12]. We took into account the potential impact of sialidase inhibitor treatment on anti-GPIIbIIIa-mediated ITP, as some anti-GPIIbIIIa antibodies can also lead to platelet desialylation.

MicroRNA (miRNA) is a single-stranded, small, non-coding RNA molecule that plays a variety of important roles in various biological processes through the post-transcriptional regulation of gene expression. Thus, it can act as a modulator of the immune system and can play an important role in the development of several autoimmune diseases [15]. Several studies have suggested that miRNAs are involved in the pathogenesis of ITP, because they can interact with the function of adaptive and innate immune responses [16]. The relationship between the mechanism of action of miRNA and ITP is complex and still only partially known. The results of many studies indicate the abnormal expression of miRNAs such as miR-155, miR-146, mir-142, and miR-181 in ITP. Chang et al. demonstrated, for example, that the level of MiR-155 is increased in ITP [17]. The detection of platelet-related miRNA can be used as a potential biomarker for the diagnosis and prognosis of ITP, as well as to predict patients’ responses to TPO-RA treatment [16].

### 4.3. Risk Factors Associated with the Development of Chronic ITP

In ITP the underlying mechanisms are heterogeneous and the DE sialylation clinical manifestations are highly variable. Patients with ITP at the same platelet level can experience varying degrees of severity of bleeding, including cerebral bleeding. ITP is not just a single entity, but rather a group of disorders of different causes.

The diagnosis of the disease is based on the presence of thrombocytopenia with a normal white blood cell count and normal value of hemoglobin, where other secondary causes of thrombocytopenia are excluded. Patient management must estimate the risk of bleeding, a complication that can occur in patients with ITP and which implies a rational and current approach to evidence-based treatment, such as the recommendations present in the recent guidelines of the American Society of Hematology (ASH) [18]. Since up to 21% of patients with ITP are asymptomatic at diagnosis, it is important to establish the criteria for the initiation of treatment. Several studies have shown that patients with ITP with platelet counts persistently < 30 × 10^9^/L are at life-threatening risk for bleeding. In general, most doctors decide to start treatment when the platelet count falls below this threshold [8].

Being over 10 years old at diagnosis, a platelet count between 10 and 20 × 10^9^/L, an absence of mucosal bleeding, an absence of viral infection at onset, and the presence of antinuclear antibodies (ANAs) have been reported as risk factors for chronicity [18]. In a study conducted by Gungor et al. on a group of 209 children diagnosed with ITP, the average age at the onset of the chronic form was 6.8 years (+/−4.2 years) compared to the average age at the onset of the acute form of the disease of 4.8 years (+/−3.9 years) [19].

The same age of onset was also confirmed in the cohort study by Glanz et al., who noted that patients who were diagnosed after the age of 10 and had a platelet count above 20,000/mmc had a 5-times greater risk for chronicity compared to patients under 2 years of age with a baseline platelet count below 20,000/mmc [20]. Another multicenter study on a group of 1683 children diagnosed with ITP reported a higher probability of acute ITP for children under 1 year of age, whereas children aged 1 to 9 years showed similar percentages for the acute and chronic form of the disease, and children aged over 9 years at diagnosis presented a higher risk of developing chronic ITP (51% of cases) [20]. Revel-Vilk et al. [21] in their study of 472 children, developed a prediction rule for the risk of chronic ITP in children younger than 10 years and with a history of bleeding for less than 2 weeks at diagnosis. High-risk children were over 10 years of age and had bleeding symptoms for more than 2 weeks at diagnosis, concluding that only 11% of children with low-risk ITP develop chronic ITP compared with 63% of high-risk children at diagnosis [22]. In our study, the statistical analysis showed that the remission rate was higher for patients aged between 2 and 12 months with a platelet count at onset below 10 × 10^9^/L, compared to the age group of 9–18 years with a platelet count at onset below 10 × 10^9^/L. Additionally, for the age group of 2–12 years with an initial platelet count more than or equal to 10 × 10^9^/L, the remission rate was double compared to the age group of 9–18 years with an initial platelet count below 10 × 10^9^/L. Several recent studies demonstrated that the age at onset appears to be the most important prognostic factor. According to data from the literature, an age less than 1 year offers the most favorable prognosis of the disease [22,23].

Schmidt et al. developed a tool for predicting the evolution of the disease on a group of 571 children with ITP (two cohorts, the NOPHO cohort and the Tiki Trial). The following predictor factors were included: age, sex, previous history of infection, insidious onset, platelet count at onset, presence of wet purpura, and calculating The Childhood ITP Score. This score can be calculated online (http://www.itprecoveryscore.org, accessed on 4 October 2022) [24].

Regarding the gender distribution, in our study 35% of the female patients presented with the chronic form of the disease compared to 21% of the male patients. The risk of having multiple hospitalizations for a female patient with the chronic or persistent form of the disease was 2.8 times higher than for a male patient; these data were consistent with those in the recent literature [25].

Patients with a history of infection showed a lower risk of chronicity. The mean platelet count at baseline for patients with the acute form of the disease was 9.4 +/− 12.4 × 10^9^/L, compared to a mean value of 17.5 +/− 19.4 × 10^9^/L for patients with the chronic form of the disease [19].

Li et al. showed that the absolute lymphocyte count (ALC) at the time of diagnosis is an independent factor leading to chronic ITP in children [26]. When an ALC of 3.005 × 10^9^/L was used as the cutoff value, the non-remission rate was found to be statistically different. Wang Yongxin et al. showed that chronic ITP and non-chronic ITP forms differ significantly in terms of the ALC [27]. The initial level of the ALC in children with chronic ITP is lower than in children with non-chronic ITP [28]. This is consistent with the results of the study by Sun Y et al. Logistic regression analysis found that when the ALC is >4.905 × 10^9^/L, the possibility of childhood ITP progressing to chronicity is very low, and thus the ALC is an independent predictor of chronic ITP [29]. In our study group, 100 patients developed chronic/persistent ITP, and approximately half of them had associated lymphopenia of 3.005 × 10^9^/L at the onset of the disease.

A study on a cohort of 409 children by Edslev et al. [30] aimed to identify and develop a tool to help predict the disease outcome in children with ITP who had an evolution of more than 3 months. The clinical characteristics that were included in the study were: abrupt onset of the disease, age at onset under 6 years, recent infection or previous vaccination, a platelet count below 5 × 10^9^/L, mucosal bleeding, and male sex. The Nordic score was validated in two different cohorts of children. High scores (10–14) clearly identified low-risk patients. The Nordic score provides valid prognostic information. Children with high scores, in whom the period of severe thrombocytopenia is unlikely to last more than 1 month, could be considered for a no-treatment approach, and children with low scores could be prepared for prolonged follow-up [22]. The impact of the Nordic score on clinical practice was also validated by other studies, such as the study on a cohort of 308 patients by Zeng et al. who concluded that it is an independent predictor of the resolution, recurrence, and development of chronic ITP [24].

A meta-analysis from 2014 by Heitink-Pollé et al. reviewed over 50 selected articles to highlight risk factors associated with the development of chronic ITP. The results showed that female sex, an age over 11 years at onset, the absence of infection or vaccination, insidious onset of ITP, a higher platelet count (≥20 × 10^9^/L), positive antinuclear antibodies, as well as combined treatment with IVIG and methylprednisolone are indicators for the chronic evolution of ITP. Bleeding as a predictor factor of chronic evolution is controversial, with findings differing from one study to another in the analyzed studies [31].

Additional or concurrent infections can influence the progression of ITP to its chronic form. In fact, in children, ITP is often considered a post-infectious sequel. There may also be an underlying asymptomatic viral illness that eludes detection. It is unclear whether all ITP patients should be tested for hepatitis C and human immunodeficiency virus. Primary treatments for both hepatitis C and human immunodeficiency virus increases platelet counts. However, if hepatitis C is detected in the cirrhotic stage, the platelet effects of the antiviral treatment may be limited [32]. The novel coronavirus also has an impact on immune thrombocytopenia. Like other viruses, COVID-19 can be responsible for ITP with a transient resolution, but it can also trigger a breakdown of tolerance that can lead to persistent or chronic ITP [33]. Another subclinical viral infection is cytomegalovirus infection, which may only be revealed by atypical lymphocytes and/or mildly elevated liver tests [34]. Cytomegalovirus or Epstein–Barr virus can exacerbate ITP in patients receiving immunosuppressive treatments, as these agents will activate the virus and thus worsen ITP, making it more resistant to treatment [34,35]. *Helicobacter pylori* may be a “cause” of ITP in certain areas (Japan, and Italy), but its eradication seems to be a uniformly effective approach to chronic ITP [34].

### 4.4. Therapeutic Regimen in Chronic ITP

In the majority of children with ITP, the disease is gone within six months with or without treatment. However, about 20–30% of patients may develop the chronic form of the disease, which may last more than 12 months. Some of these chronic patients may be refractory to first-line therapy (corticosteroids, immunoglobulin, and anti-D globulin) and may experience severe, even life-threatening, bleeding [36]. In such cases, the second line of therapy is either immunosuppressive therapy with rituximab or splenectomy [37]. However, some patients may continue to be refractory to second-line treatment and may continue to experience severe bleeding episodes. In our study group, 62.0% of patients received corticosteroid therapy, 24.7% received a combination of corticosteroid therapy and intravenous immunoglobulins, and 3.7% were treated only with IVIG. Only 1.5% of patients required splenectomy, and 8.1% did not receive treatment. A study of a cohort recently published by Elkus et al. collected retrospective data from seven centers comprising 205 patients diagnosed with ITP. Most patients received initial drug therapy, including intravenous immunoglobulin (54%), corticosteroids (9%), and anti-D (3%), whereas 37% were only followed [38]. In the 6 months after diagnosis, 11% were readmitted to hospital for management of chronic ITP [37]. The study of Bennet et al. also noted that most children with immune thrombocytopenic purpura have a favorable outcome with initial therapy. A small but significant minority develops chronic ITP, requiring continuous therapy regimens [35]. In such cases, thrombopoietin (TPO) receptor agonists such as romiplostim and eltrombopag are used to increase the production of platelets in the bone marrow. They produce a sustained increase in platelet count and are approved for use in ITP patients in the US, Europe, and Japan [39]. In 2010, thrombopoietin receptor agonists were licensed as a second-line treatment for chronic ITP [40]. It was found that 40% of children with chronic ITP responded to eltrombopag therapy [41]. In 2017, rituximab (RTX) as a second-line therapy was also licensed for patients, including children with chronic ITP [42]. However, only 14–26% of children had a prolonged response with platelet counts above 100 × 10^9^/L [42]. The occurrence of complete remission in children with chronic ITP under IVIG therapy, glucocorticoids, or splenectomy was 30–52% at five years [42].

Therapy for children with refractory ITP remains a challenge and suitable treatment approaches remain debatable. At the time of diagnosis, it is not possible to differentiate between patients who will have a short evolution of thrombocytopenia compared to those who will develop the chronic disease. Predictors of ITP evolution would help guide treatment decisions and also allow us to improve the quality of life of patients with the chronic form of ITP [35].

## 5. Conclusions

An age at diagnosis over 9 years and female sex represent the main risk factors for chronicity. A platelet value above 10 × 10^9^/L at onset may be associated with an increased risk of developing persistent/chronic forms of the disease, but further studies on large groups of patients are needed to validate a consensus.

## Figures and Tables

**Table 1:** Recovery rate in relation to presenting platelet count.

Age Group	Platelet Count < 10 × 10^9^/L	Platelet Count ≥ 10 × 10^9^/L	95% CI for Difference
2–12 months	10/12 (83.3%)	22/26 (84.6%)	14.2 to 11.6
1–8 years	51/68 (75.0%)	40/72 (55.6%)	11.6 to 27.3
9–18 years	23/41 (56.1%)	25/52 (48.1%)	2.4 to 18.4
Total	84/121 (69.4%)	87/150 (58.1%)	5.6 to 17.2

**Table 2:** The evolution of ITP based on the chosen therapy.

		Treatment			
Recovered		No Treatment	Corticosteroids	Corticosteroids + IGIV	Total
NO	Observed	13	51	27	91
	% within row	14.3%	56.0%	29.7%	100.0%
YES	Observed	9	117	40	166
	% within row	5.4%	70.5 %	24.1%	100.0%
Total	Observed	22	168	67	257
	% within row	8.6%	65.4%	26.1%	100.0%

**Table 3:** Kruskal–Wallis Test.

	χ^2^	df	*p*
RECOVERED	7.939	2	0.019

## Data Availability

The data that support the findings of this study are available upon request from the corresponding author.
